# The cancer patient’s wife

**DOI:** 10.3390/curroncol13060021

**Published:** 2006-12

**Authors:** Mark Handelman

Mrs. Lee, it’s your husband’s nurse calling from the hospital. We need to know now whether you’ll want us to resuscitate Mr. Lee, just in case.

A friend of mine, Mary, received this call yesterday. Her husband Tom had been hospitalized a week earlier, after his platelet count dropped so low that daily outpatient blood transfusions became inadequate and other symptoms of his terminal cancer made living at home impossible.

I wondered about the timing and appropriateness of the call. Absent a sudden change in Tom’s condition, he was still capable of making his own treatment decisions. Besides, Mary had camped out in the hospital since Tom’s admission. She went home for only a few hours each day to shower and relax, during which time one of their children took over the vigil. To a woman coming to grips with her husband’s terminal condition, such a call could only mean the worst.

It turned out that the call was somewhat routine: a nurse had noticed that the section of Tom’s chart detailing resuscitation was incomplete and had decided to call Mary for instructions. We will never know why she couldn’t wait for Mary’s return to the hospital.

But for me, other questions arose.

Tom’s mental capacity was not yet diminished—neither by the disease he suffered nor by pain medication. Why wasn’t the nurse asking him, instead of Mary, for his end-of-life wishes?

Tom had been in hospital more than a week. Wasn’t some sort of protocol in place for determining the answers to these questions, at least for patients whose conditions were likely terminal?

In most of the developed world and much of the developing world, one guiding ethical principle of the relationship between a patient and his or her treatment team is respect for the dignity and autonomy of the patient. In Ontario that principle is codified in both the *Health Care Consent Act* and the rules of professional conduct for each of the health care colleges.

Capable patients have the right to make their own treatment decisions and to express advance wishes for treatment in anticipation of incapacity. Substitute decision-makers for incapable patients have the obligation to follow those previously expressed wishes provided they were made when the patient was capable and are applicable to the circumstances of the treatment decision. It is also easier on the patient’s family and the treatment team alike to know in advance the decisions a patient would make at the end of life.

Another guiding ethical principle of treatment is that decisions are based on *informed* consent. I have the sense that most people in North America get most of their medical information from television. To them, doctors are magicians. When a heart stops, the doctors and nurses rush into a patient’s room and wave a magic wand. Then, after the next commercial, the patient is discharged, fully recovered. Most people don’t know about the physical damage that resuscitation inflicts on a frail body, don’t know the extent to which cpr is likely futile.

Tom and Mary are not sophisticated. Their discussions with Tom’s treatment team about his palliative care haven’t gone much further than “Do everything you can to keep him alive.” Is it my obligation or the treatment team’s to explain to them what such an instruction actually means?^a^

Please don’t think me arrogant for pontificating to dedicated, overworked professionals trying to save lives and ameliorate pain. I wrote this not only out of concern for a friend and his wife, but also because I’ve seen the moral distress that these dedicated professionals experience when their patient’s suffering is extended and exacerbated by treatment decisions born of unrealistic expectations and inadequate information.

Your comments and contributions to this column are welcome.

## Footnotes

a The Web site of the University of Toronto’s Joint Centre for Bioethics (www.utoronto.ca/jcb/outreach/documents/JCB_Living_Will_Cancer.pdf) offers an outstanding resource that addresses the end-of-life decision-making process for cancer patients. It explains many legal and medical terms in plain English.

